# Wisconsin State Medical Society

**Published:** 1869-01

**Authors:** 


### WISCONSIN STATE MEDICAL SOCIETY.

The meeting of the State Medical Society convened at the State Agricultural Rooms at 4 P.M., June 10th, 1868. Dr. H. Van Dusen, President, in the chair. Dr. H. P. Strong, Secretary, not being present, Dr. A. J. Ward, of Madison, was appointed temporary Secretary.

On motion of Dr. Brown, Drs. Carr and Dalton were appointed, *pro tem.*, to act as Censors, in place of those absent.

### CREDENTIALS OF APPLICANTS EXAMINED.

Those presented were: Isaac E. Thayer, Brandon, Fond du Lac Co., graduate of Rush Medical College, also of University of Illinois; Antinous A. Rowley, Middleton, Dane Co., Rush Medical College; A. H. Salisbury, Mazo, Maine, graduate of Bellevue Hospital Medical College; I. J. Whitney, Prairie du Chien, graduate of Buffalo Medical University; vouched for by Drs. Mason and Favill. L. G. Armstrong, Boscobel, graduate of Rush Medical College; vouched for by Drs. Mason and Corey. D. M. Bond, Johnstown, Rock Co., graduate of Chicago Medical College; James Cody, Watertown, Jefferson Co., graduate of Harvard, Mass.; Henry M. Lilley, Fond du Lac, Wis., graduate of Medical Department of the University of Michigan; vouched for by Dr. Isaac E. Thayer, Brandon.

On motion of Dr. Taggart, of Beloit, the above-mentioned gentlemen were duly elected members of the Society.

On motion of Dr. Favill, the new members were allowed all the privileges of the Society in the discussions that may arise before the arrival of the Secretary and Treasurer.

There was some discussion as to whether the Society would grant diplomas, and, if so, on what basis.

On motion of Dr. Marks, adjourned to 7½ o'clock.

7½ P.M.

Reports of Standing Committees were passed over until tomorrow morning.

Dr. Marks moved to adjourn, which was lost.

Dr. Taggart moved that miscellaneous business be taken up. Carried. He also suggested that some action should be taken with regard to examinations for life insurance.

Dr. Cody suggested that something should be done, in petitioning the Legislature, with regard to services as experts in medical testimony.

On motion of Dr. Marks, it was resolved that a committee of three be appointed by the President, to ask such legislation as they may think proper on the subject. Carried.

Dr. Marks moved that Dr. Strong receive from the Society $25, to pay his clerk-hire, which was carried.

Drs. Cody, Carr, and Barrett were appointed by the Chair, to ask some legislation for pay as experts, in giving medical testimony.

Dr. Whiting moved that another committee be added to our list—which comes under the head of *Mental and Nervous Diseases*. Carried. Drs. Van Nostrand, Stoddard, and Corey were appointed on that committee.

Dr. Cody reported a case of fracture of the ribs. The interesting part of the case was, the extensive emphysema that ensued—it having extended over the whole body. There was also extensive hemorrhage of the lung.

Dr. Whiting spoke of the danger of fracture of the ribs; did not believe that in half of the cases reported there was any fracture.

Dr. Taggart thought the danger depended upon the displacement of the bone.

Dr. Barrett said he had known of 10 or 12 cases, that had been reported in his town, and he did not know of but two of the cases that were really fractured.

The Censors then made the following report:—

"Your Censors, having examined Dr. Mayham, concur in recommending him for membership, as much upon the endorsement of his neighboring practitioners as upon the result of the examination. We would further state, we cannot recommend him for a diploma.

[Signed,] "N. DALTON,
"JAS. BROWN,
Adopted. "*Censors.*"

On motion, adjourned until 8 o'clock to-morrow morning.

A. P. WARD, *Sec'y, pro tem.*

June 11, 1868, 8 o'clock A.M.

Meeting called to order by President Van Dusen. Present —Drs. Van Dusen, Strong, Ferrin, Brown, Favill, Ward, Thayer, Carr, Taggart, Dickson, Mason, Marks, Dalton, Corey, Stoddard, Whiting, Barrett, Ellsworth, Benson, Linde, Coolidge, Cody, Armstrong, Whitney, Bond, McKennan, Lilly, Mayham, Millard, N. C. Rowley, A. A. Rowley, Jones, and President Chadbourne.

Gov. Fairchild presented an invitation to the gentlemen of the Medical Society to spend the hour of 7 to 8 o'clock at his house, this evening. Accepted.

Minutes of last meeting read and approved. Report of Secretary accepted.

Dr. Brown offered the following resolution, *viz.:*—

*Resolved*, That the Board of Censors having examinod the qualifications of Dr. T. F. Mayham, and reported favorably thereon, he be admitted to membership in this Society.

Dr. Favill moved that the Censors have leave to withdraw their report on the case of Dr. Mayham, made last evening. Adopted.

On motion of Dr. Favill, the resolution offered by Dr. Brown was then accepted as a report of the Censors.

Moved, by Dr. Taggart, that Dr. Mayham be elected a member of this Society. Motion lost.

Moved, by Dr. Strong, that the rules and regulations relative to the admission of members be suspended during action on the case of Dr. Mayham, and that hereafter the Censors are instructed not to entertain any application for membership, unless the applicant shall conform to the rules then existing as to admission to membership in this Society. Adopted.

On motion of Dr. Ward, the motion rejecting Dr. Mayham was reconsidered.

On motion of Dr. Ward, he was then elected a member.

Dr. Brown then offered the following resolution, which was adopted:—

*Resolved*, That in explanation of the action of the Society heretofore upon the subject of admission to membership, the following conditions are required:—The applicant shall present to the Board of Censors a diploma from a regular school of medicine, of good repute; or a certificate or diploma of membership in a county or district society, accompanied with a certificate of six years' practice, and of good moral and professional character; or he shall submit to such an examination as the Censors may impose.

Censors reported in favor of admitting Dr. F. R. Millard, of Beetown, graduate of Rush Medical College, to membership. Report adopted, and he was elected a member.

Dr. Ward asked leave to present a patient to the Society for opinion as to the nature of the disease, which was granted, and an examination made by the members of the Association present. The patient was a lady of about 30 years of age. After confinement some time since, she left her bed rather too early, and was attacked with roaring in the right side of the head. This is constant, and very annoying. Instant relief is obtained by pressure upon the common carotid artery, and remains so long as the pressure continues. This is the only means by which relief is afforded. There was a diversity of opinion as to the nature of the difficulty, and no definite conclusion obtained.

Dr. Marks, Chairman of the Committee on Surgery, made a report at length on treatment of fractures of the thigh. The report goes on to prove, by the best authority, that oblique fractures of the femur in adults nearly always results in more or less shortening. He also proved that the majority of surgeons prefer the straight position in treatment of these fractures; and goes on to say, that, "when there is displacement of the fragments, the points of bone must be in contact with the soft parts, and that any unnecessary handling or twisting of the limb inflicts injury upon the muscles, and other tissues, which taxes nature to repair. Gentleness is not incompatible with good surgery, although there are men who, judging from their method of manipulation, would differ with me. Those claiming to be good surgeons, often inflict an amount of unnecessary injury to the soft parts, in their examination, that takes nature weeks, I might say, months, to repair." The treatment he has adopted and prefers is to use extension by means of adhesive plaster applied to the leg, terminating below the foot, to which is attached a weight running over a pulley, varying in size from 8 to 20 pounds in adult cases. A sand-bag is placed on each side of the limb, and the foot of the bedstead elevated 3 or 4 inches, the weight of the body being the counter-extension.

When the fracture occurs below the lesser trochanter, he applies pasteboard splints the whole length of the limb, upon either side—using the extension as before indicated.

"The advantages claimed for this method are:—First, ease and comfort to the patient. Second, the dressing does not interfere in the least with the circulation in the limb. Third, the limb can be seen at all times; and, in case of compound fracture, the wound can be dressed as often as desirable. Fourth, the limb can be dressed and placed in position in one-fourth the time required to apply the splint and roller. Fifth, I claim better results from this method than from any other I have ever seen tried, though I do not pretend that it will always prevent shortening."

In fractures within the capsule, or partly within, he uses only sand-bags, with the weight and pulley, as before alluded to.

Dr. Marks gave several cases treated in this manner; and the result warrants a general trial.

Dr. Mason, of Prairie du Chien, was called upon to give the result of his experience in the treatment of fractures without splints.

He made a statement, that he had treated fractures of the femur and tibia by the use of sand-bags and the weight, with good success; and had also used the same treatment for synovitis, with good results.

Dr. Marks reported at the previous meeting a case of aneurism of the subclavian artery, then under treatment, and for which he had amputated at the shoulder-joint. The patient recently died, and he presented a full written report of the case, with a statement of the appearance of the tumor after death.

Dr. McKennan, of Sauk City, presented a written report of a case of shoulder presentation, where the uterus was ruptured during the act of version. The child was immediately delivered, and the mother recovered.

He also presented the written report of another case, where the woman was very short of stature, but stout and robust, and had a deformity of the pelvis, from an undue prominence of the promontory of the sacrum. Antero-posterior diameter less than three inches. She had borne twelve children, nine of which had been delivered with instruments, and were dead. The 10th, 11th, and 12th were very small, and were born without instrumental aid. He was called to her in her 13th confinement, and found the pains strong, and the head presenting, and pressing strongly against, and partially engaged in, the brim of the pelvis; and so it remained for 12 hours. The Doctor then turned the child, and delivered it by the feet—considerable force being required to extricate the head. The child was alive, and weighed some ounces over 13 pounds.

The report of this case gave rise to some discussion as to the practicability of turning in such cases—some apprehending that in most cases the head could not be extricated.

Dr. Dalton, of Mineral Point, made an extended report of a case of aneurism of the axillary artery, which terminated in death.

The patient presented himself "with a large pulsating tumor in the left axilla, a small part presenting above and within the acromial articulation of the clavicle, the scapula considerably elevated, a pain, at times, in the left arm, with considerable swelling, and at all times a numbness, or, as he expressed it, a 'dead-feeling, extending to the ends of his fingers.'" He goes on to give a history of the case from the first appearance of the tumor. Told the patient the only remedy was ligation, and that the result would probably be fatal under any method of treatment. He decided to have the operation performed, and Dr. Dalton tied the left subclavian artery, at the outer margin of the external scalenus muscle, following the form of incision introduced by J. Kearney Rodgers.

The operation was followed by sensible diminution in the size of the tumor, and coldness of the whole surface of the arm. Case did well up to the 24th day, when the ligature came away, followed by slight hemorrhage. Incision had entirely healed, except that portion about the ligature—warmth nearly restored, and tumefaction almost disappeared.

The case went on until the incision entirely healed, and in six days he was discharged as cured.

The operation was performed in November, and on the 7th day of March following, he felt pain in the back and right shoulder, and on the night of the 10th he suddenly expired.

Dr. Dalton closes his report, which is given in detail, as follows:—

"I requested a *post mortem*, but could not prevail upon them to grant it. Therefore, of what he died I am wholly ignorant, but, from their description, came to the conclusion that it might have been angina pectoris. They stated he had been suffering, as usual, with his pain, when he suddenly threw his right hand to his left side, and screamed with pain; turning himself partially over, he became some easier, but his ease was of short duration. In from three to five minutes he acted in the same manner, and expired.

"Whether this could in any way be connected with the aneurism or operation, is a question I leave to the profession to decide."

Adjourned to 1½ o'clock.

1½ o'clock.

The Committee on Practical Medicine was not ready to report, and was continued, with the exception of the Chairman. Dr. C. J. Taggart, of Beloit, was appointed Chairman.

Dr. Ferrin, of the Committee on Practical Medicine, made a verbal report of an anomalous case of pain and irritability of the urethra.

The subject of puerperal convulsions having been incidentally brought up, a very general and animated discussion upon the pathology and treatment followed. President Van Dusen left the chair and participated, in his usual pointed and energetic style; he was full of the subject. Being an elderly gentleman, of large experience and close powers of observation, he was pretty good authority upon this subject. His opinion was, that it usually occurred in persons of plethoric or full habit, and bleeding was the remedy. Such had been his practice, and he had never lost a case. He had but little faith in anæsthetics without bleeding, and such appeared to be the general opinion.

Most of the afternoon was consumed in the report of cases and general discussion.

President Chadbourne, of the State University, appeared, and was introduced by President Van Dusen. He gave some practical remarks upon the duties and requirements of the medical profession. He introduced the subject of establishing a medical school within the State under the law creating the University, and sai., as one of the members of the committee appointed by the Regents to take into consideration the practicability of a school to be established at Milwaukee or elsewhere, he felt desirous to advance the project, but he felt that the initiative should be taken by the State Medical Society, as it must in a great measure depend upon it for its support and direction, and he hoped that the Society would take action upon this when it seems to be desirable to organize such a school.

The subject of treatment of rheumatism was under discussion for some time, and the majority seemed to favor alkalies as the most efficacious remedies.

On motion of Dr. Marks, of Milwaukee, Dr. Taggart, of Beloit, was requested to furnish a written report of a case of spontaneous rupture of the sphincter ani; also, that Dr. McKennan, of Sauk City, be requested to furnish a written report of his treatment of fracture of the malar bone; and Dr. Cody, of Watertown, of a case of emphysema.

Dr. Van Dusen announced the death of Dr. J. B. Dousman, and moved that a committee be appointed to give a biographical sketch of Dr. Dousman at the next meeting. Adopted. Dr. Whiting, Vice-President, in the chair, appointed Dr. H. Van Dusen.

On motion, Drs. Jesse Moore, of Beloit, and Azariah Blanchard, of Milwaukee, were elected honorary members.

On motion, a standing committee of three was appointed to report on new remedies. Chair appointed Drs. Cody, Corey, and Mason.

On motion of Dr. Whitney, of Prairie du Chien, a committee on diseases of the eye and ear was appointed. Chair appointed Dr. Whitney.

Dr. Mason presented a biographical sketch of Dr. B. F. White, deceased, and a report was presented by Dr. Treat on the life of Dr. C. S. Farr, deceased.

A communication from Dr. Storrs Hall, of Rosendale, was presented, regretting his inability to attend.

On motion of Dr. Marks, the Secretary was instructed to furnish the Chicago medical journals with a synopsis of the proceedings.

Moved, by Dr. Marks, that the Constitution, By-Laws, and Code of Ethics, with the Proceedings, be published in pamphlet form, and distributed to the physicians generally in this State. Adopted.

Dr. Whiting presented the following resolution, which was adopted:—

*Resolved*, That whenever any member shall have neglected to pay his annual dues to this Society for the period of three years, his name shall be stricken from the rolls of the Society, in disgrace; provided, however, that the Secretary shall previously send him a copy of this resolution.

Dr. Dalton offered the following:—

*Resolved,* That when we adjourn we adjourn to meet on the 3d Wednesday of June next, at the city of Madison. Adopted.

Dr. Barrett offered the following:—

*Resolved,* That in view of the increasing population of Wisconsin and other Northwestern States, a larger number of well and thoroughly educated medical men are demanded to meet the wants of the community, the time has come for the establishment of a medical school in this State, under the auspices of the State Medical Society.

*Resolved,* That a committee of three be appointed to confer with a similar committee recently appointed by the regents of the State University, in regard to the immediate establishment of such a school.

Laid over to next meeting.

A committee of one from each Congressional District to furnish the Secretary the names of physicians in the State, was appointed, as follows:—

*First District*—Dr. Marks, Milwaukee.

*Second District*—Dr. Favill, Madison.

*Third District*—Dr. Dalton, Mineral Point.

*Fourth District*—Dr. Lilley, Fond du Lac.

*Fifth District*—Dr. Russell, Oshkosh.

*Sixth District*—Dr. Thayer, Brandon.

Moved by Dr. Strong, that a committee on Pathology and a committee on medical education be appointed. Adopted,

The Chair announced the standing committees for the ensuing year as follows:—

*Arrangements*—Drs. Brown, Favill, and Ward.

*Surgery*—Drs. Marks, Dalton, Palmer.

*Obstetrics*—Drs. McKennan, Barrett, Thayer.

*Pathology*—Drs. Stoddard, Bond, Armstrong.

*Practice*—Drs. Taggart, Vivian, Ferrin.

*New Remedies*—Drs. Cody, Corey, Mason.

*Executive Committee*—Drs. Carr, Dickson, Salisbury.

*Medical Education*—Dr. Carr.

The thanks of the Society were tendered to Gov. Fairchild for the hospitality of this evening.

Adjourned. H. P. STRONG, *Secretary.*

